# Impaired glucose metabolism is associated with increased thrombin generation potential in patients undergoing angioplasty and stenting

**DOI:** 10.1186/s12933-018-0774-0

**Published:** 2018-09-29

**Authors:** Silvia Lee, Cihan Ay, Christoph W. Kopp, Simon Panzer, Thomas Gremmel

**Affiliations:** 1 Department of Internal Medicine II, Medical University of Vienna, Waehringer Guertel 18-20, 1090 Vienna, Austria; 2 Department of Internal Medicine I, Medical University of Vienna, Vienna, Austria; 3 Department of Blood Group Serology and Transfusion Medicine, Medical University of Vienna, Vienna, Austria; 4 Department of Internal Medicine, Cardiology and Nephrology, Landesklinikum Wiener Neustadt, Wiener Neustadt, Austria

**Keywords:** Thrombin, Thrombin generation potential, Diabetes, HbA1c, Angioplasty, Stenting

## Abstract

**Background:**

As a strong platelet agonist on the one hand and key molecule in plasmatic coagulation on the other hand, thrombin connects primary and secondary hemostasis. Thrombin generation potential reflects the individual capacity to generate thrombin, and has been associated with the occurrence of thromboembolic events. In the current study, we sought to identify predictors of thrombin generation potential in patients undergoing angioplasty and stenting for atherosclerotic cardiovascular disease.

**Methods:**

Peak thrombin generation potential and area under the curve (AUC) of thrombin generation potential were determined with a commercially available assay in 315 patients on dual antiplatelet therapy 1 day after percutaneous intervention, and in 100 healthy individuals without cardiovascular disease.

**Results:**

Median (interquartile range) peak thrombin generation potential and AUC of thrombin generation potential in the study cohort (n = 315) were significantly higher than in healthy individuals (n = 100) without cardiovascular disease (peak thrombin generation potential: 445.4 nM [354.5–551.8 nM] vs. 174.5 nM [141.2–261.2 nM]; AUC of thrombin generation potential: 5262.7 nM thrombin [4806.6–5756.9 nM thrombin] vs. 3405.2 nM thrombin [3043.6–3747.3 nM thrombin]; both p < 0.001). In patients undergoing angioplasty and stenting, hemoglobin A1c (HbA1c) was the only variable that was independently associated with both, peak thrombin generation potential and AUC of thrombin generation potential (both p ≤ 0.007). In contrast, platelet count and high-sensitivity C-reactive protein were only associated with peak thrombin generation potential, and body mass index and serum creatinine were only associated with AUC of thrombin generation potential after adjustment for covariates by multivariate linear regression analyses (all p < 0.05). Patients with HbA1c ≥ 6% had significantly higher peak thrombin generation potential and AUC of thrombin generation potential than patients with HbA1c < 6% (peak thrombin generation potential: 476.9 nM [385.8–577.9 nM] vs. 423.9 nM [335.8–529.5 nM], p = 0.002; AUC of thrombin generation potential: 5371.8 nM thrombin [4903 – 5899 nM thrombin] vs. 5172.5 nM thrombin [4731.8–5664.7 nM thrombin], p = 0.01). HbA1c ≥ 6% remained independently associated with both parameters of thrombin generation potential after multivariate linear regression analyses (both p ≤ 0.02).

**Conclusions:**

Impaired glucose metabolism is associated with increased thrombin generation potential in patients undergoing angioplasty and stenting for cardiovascular disease.

## Introduction

Patients with atherosclerotic cardiovascular disease are at an increased risk of ischemic events such as myocardial infarction and stroke, which are the main causes of death in high income countries. Previous studies by others and us revealed higher in vivo expression of platelet surface P-selectin and activated glycoprotein IIb/IIIa as well as increased levels of monocyte-platelet aggregates in these patients compared to healthy individuals [1–3]. P-selectin and activated glycoprotein IIb/IIIa are expressed on the surface of activated platelets and can be measured by flow cytometry [4, 5]. Monocyte-platelet aggregates are mainly formed via the interaction of platelet surface P-selectin with its counterreceptor P-selectin glycoprotein ligand-1 on leukocytes [6], and were shown to be an even more sensitive indicator of ongoing platelet activation than P-selectin in several pathophysiological circumstances including myocardial infarction [7]. Besides these cellular markers, various soluble parameters of platelet activation are elevated in patients with cardiovascular disease [8, 9]. In particular soluble P-selectin deriving from platelet α-granules and endothelial Weibel-Palade bodies [6, 10], is increased in advanced atherosclerosis [8], and has been linked to the occurrence of ischemic outcomes [11]. Altogether, the above-mentioned findings point towards a smoldering prothrombotic state in cardiovascular disease.

In this environment, the exposure of subendothelial structures of the vessel wall due to the rupture of an atherosclerotic plaque leads to immediate platelet adhesion to collagen fibers [12]. Platelets become activated, release the content of their granules and trigger the recruitment of further platelets and the activation of the hemostatic system, which entails local thrombin generation [6, 12, 13]. As a strong platelet agonist on the one hand and key molecule in plasmatic coagulation on the other hand, thrombin connects primary and secondary hemostasis. Thus, it plays a pivotal role in the processes resulting in intravascular thrombus formation with narrowing or even occlusion of the affected artery and subsequent end organ ischemia [14]. In detail, thrombin rapidly activates human platelets via protease-activated receptors (PAR)-1 and PAR-4 even in the presence of state-of-the art antiplatelet therapy [15, 16]. It propagates platelet adhesion and aggregation, and thereby the formation of a first platelet plug [6]. As a serine protease, thrombin subsequently converts fibrinogen to fibrin resulting in the stabilization of the thrombus [14]. Recent data suggest that the formation of denser fibrin clots displaying an impaired lysability may be associated with worse clinical outcomes in patients with acute ischemic stroke [17]. Besides its role in thrombosis and hemostasis, thrombin serves as a signalling molecule and activates cell complexes involved in the development and progression of atherosclerosis [18].

Thrombin generation potential reflects the individual capacity to generate thrombin, and can be quantified by a commercially available assay [19]. Measuring thrombin generation potential may offer a way to identify patients with a hyper- or hypocoagulable phenotype [20]. Indeed, it has been shown that increased thrombin generation potential is associated with the occurrence of venous thromboembolism (VTE) in cancer patients and with recurrence of VTE after a first unprovoked thromboembolic event [21, 22]. Attanasio et al. found thrombin generation potential to predict cardiovascular death after acute coronary syndromes [23]. Furthermore, previous studies revealed higher thrombin generation potential in patients with lupus anticoagulant, protein C and S deficiencies, factor V Leiden mutation, and elevated coagulation factors VIII, IX and XI, as well as in women taking oral contraceptives or hormone replacement therapy [24].

Given the previous reports on thrombin generation potential as risk factor for adverse cardiovascular outcomes [20–23], we sought to identify predictors of thrombin generation potential in patients undergoing angioplasty and stenting for atherosclerotic cardiovascular disease.

## Methods

### Study population

The study population consisted of 315 patients on dual antiplatelet therapy after percutaneous intervention with endovascular stent implantation. All patients received daily aspirin (100 mg/days) and clopidogrel therapy (75 mg/days). Moreover, thrombin generation potential was determined in 100 healthy controls without cardiovascular disease (65 men, 35 women; median age 47 years [39–55]).

Exclusion criteria were a known aspirin or clopidogrel intolerance (allergic reactions, gastrointestinal bleeding), a therapy with vitamin K antagonists (warfarin, phenprocoumon, acenocoumarol) or novel oral anticoagulants (rivaroxaban, apixaban, dabigatran, edoxaban), treatment with ticlopidine, dipyridamole or nonsteroidal antiinflammatory drugs, a family or personal history of bleeding disorders, malignant paraproteinemias, myeloproliferative disorders or heparin-induced thrombocytopenia, severe hepatic failure, known qualitative defects in thrombocyte function, a major surgical procedure within 1 week before enrollment, a platelet count < 100,000 or > 450,000/µl and a hematocrit < 30%.

The study protocol was approved by the Ethics Committee of the Medical University of Vienna in accordance with the Declaration of Helsinki and written informed consent was obtained from all study participants.

### Blood sampling

Blood was drawn by aseptic venipuncture from an antecubital vein using a 21-gauge butterfly needle (0.8 × 19 mm; Greiner Bio-One, Kremsmünster, Austria) 1 day after the percutaneous intervention. To avoid procedural deviations all blood samples were taken by the same physician applying a light tourniquet, which was immediately released and the samples were mixed adequately by gently inverting the tubes. After the initial 3 ml of blood had been discarded to reduce procedurally-induced platelet activation, blood was drawn into 3.8% sodium citrate Vacuette tubes (Greiner Bio-One; 9 parts of whole blood, 1 part of sodium citrate 0.129 M/l) for the measurement of thrombin generation potential.

### Thrombin generation assay

Thrombin generation was measured with a commercially available assay (Technothrombin TGA kit, Techonoclone, Vienna, Austria) on a fully automated, computer-controlled microplate reader (Bio-Tek, FL X800) and a specially adapted software (Technothrombin TGA, Vienna, Austria) using the fluorogenic substrate Z-Gly-Gly-Arg-AMC (Bachem, Bubendorf, Switzerland) according to the manufacturer’s instructions as previously described [25]. The reaction was triggered with the TGA RC low reagent, which contained 71.6 pM recombinant human tissue factor lipidated in 3.2 μmol/l phospholipid micelles (phosphatidylcholine [2.56 μmol/l] and phosphatidylserine [0.64 μmol/l]). In this assay, thrombin activity is registered in a thrombin generation curve. From this curve, the parameters peak thrombin generation potential (the maximum concentration of thrombin generation) and area under the curve (AUC) of thrombin generation potential were used. All assay kits were from the same batch and all samples were tested within the same run by the same operator. The lower limit of detection with this assay is 0 for peak thrombin generation potential and the AUC of thrombin generation potential. The intra-assay coefficients of variation were 12% and 5.5% for peak thrombin generation potential and the AUC of thrombin generation potential, respectively (n = 21). Thrombin generation potential was available for all patients.

### Statistical analysis

Statistical analysis was performed using the Statistical Package for Social Sciences (IBM SPSS version 24, Armonk, New York, USA). Median and interquartile range of continuous variables are shown. Categorical variables are given as number (%). To detect differences in continuous variables, we performed t-tests or Mann–Whitney U tests, as appropriate. Differences in categorical variables were assessed by the Chi square test. The Kolmogorov–Smirnov test was used to test for normal distribution, and variables with skewed distribution were log-transformed for regression analyses. Covariates for multivariate regression analyses were selected on the basis of univariate analyses (p ≤ 0.1), including age, sex, body mass index, hypertension, hyperlipidemia, active smoking, hematocrit, white blood cell count (WBC), platelet count, serum creatinine, high-sensitivity C-reactive protein (hsCRP), and hemoglobin A1c (HbA1c). Two-sided p-values < 0.05 were considered statistically significant.

## Results

Clinical, laboratory, and procedural characteristics of the overall study population, of patients with HbA1c < 6% and of patients with HbA1c ≥ 6% are shown in Table 1.

**Table 1:** Clinical, laboratory and procedural characteristics of the overall study population, and of patients with normal (< 6%) and elevated (≥ 6%) hemoglobin A1c (HbA1c)

Characteristics	Overall (n = 315)	HbA1c < 6% (n = 156)*	HbA1c ≥ 6% (n = 145)*	p
Demographics				
Age, years	66 (58–75)	65 (57–74)	66 (58–75)	0.4
Male sex	205 (65.1)	103 (66)	95 (65.5)	0.9
BMI, kg/m^2^	26.8 (24.2–29.7)	26.2 (23.4–28.7)	27.3 (25.1–29.7)	0.007
Medical history				
Hypertension	283 (89.8)	135 (86.5)	134 (92.4)	0.1
Hypercholesterolemia	293 (93)	144 (92.3)	136 (93)	0.6
Diabetes mellitus	102 (32.4)	11 (7.1)	85 (58.6)	< 0.001
Active smoking	132 (41.9)	76 (48.7)	53 (36.6)	0.03
Laboratory data				
HbA1c, %*	5.9 (5.6–6.5)	5.6 (5.4–5.8)	6.5 (6.1–7.2)	<0.001
Hematocrit, %	38.9 (36–42)	39.6 (36.4–42.6)	38.7 (35.4–41.4)	0.1
White blood cell count, G/l	8.4 (6.9–10.4)	8.2 (6.8–10.3)	8.7 (7–10.6)	0.3
Platelet count, G/l	208 (176–251)	207 (173–249)	210 (177–259)	0.6
Serum creatinine, mg/dl	1 (0.9–1.2)	1 (0.9–1.1)	1 (0.9–1.3)	0.2
High-sensitivity CRP, mg/dl	0.8 (0.3–1.8)	0.9 (0.3–2.1)	0.8 (0.3–1.7)	0.4
Procedure				
Stent implantation	315 (100)	156 (100)	145 (100)	1
Number of stents/patient	1 (1–2)	1 (1–2)	1 (1–2)	0.3
Medication pre-intervention				
Clopidogrel	315 (100)	156 (100)	145 (100)	1
Aspirin	315 (100)	156 (100)	145 (100)	1
Statins	300 (95.2)	149 (95.5)	139 (95.9)	0.9
ACE inhibitors/ARB	272 (86.3)	135 (86.5)	124 (85.5)	0.8
Beta blockers	216 (68.6)	104 (66.7)	105 (72.4)	0.3
Proton pump inhibitors	165 (52.4)	75 (48.1)	81 (55.9)	0.2
Antidiabetic medication	96 (30.5)	11 (7.1)	79 (54.5)	< 0.001
Oral antidiabetic medication	77 (24.4)	8 (5.1)	67 (46.2)	< 0.001
Insulin	30 (9.5)	3 (1.9)	23 (15.9)	< 0.001

Continuous data are shown as median (interquartile range). Dichotomous data are shown as n (%)

*BMI* body mass index, *CRP* c-reactive protein, *ACE* angiotensin converting enzyme, *ARB* angiotensin receptor blockers

* HbA1c was only available for 301 patients (95.6%)

Median (interquartile range) peak thrombin generation potential and AUC of thrombin generation potential in the study cohort (n = 315) were significantly higher than in healthy individuals (n = 100) without cardiovascular disease (peak thrombin generation potential: 445.4 nM [354.5–551.8 nM] vs. 174.5 nM [141.2–261.2 nM]; AUC of thrombin generation potential: 5262.7 nM thrombin [4806.6–5756.9 nM thrombin] vs. 3405.2 nM thrombin [3043.6–3747.3 nM thrombin]; both p < 0.001).

In patients undergoing angioplasty and stenting, active smoking as well as increasing WBC, platelet count, hsCRP and HbA1c were associated with higher peak thrombin generation potential in univariate analyses (all p < 0.05). In contrast, increasing serum creatinine was linked to lower peak thrombin generation potential (p = 0.03). In multivariate linear regression analysis, platelet count, hsCRP and HbA1c remained independently associated with peak thrombin generation potential (Table 2).

**Table 2:** Regression coefficients (B), confidence intervals (CI), and p-values of multivariate linear regression analyses of active smoking, log-transformed platelet count (logPLT), log-transformed white blood cell count (logWBC), log-transformed serum creatinine (logCrea), log-transformed high-sensitivity C-reactive protein (logHsCRP) and log-transformed hemoglobin A1c (logHbA1c) for peak thrombin generation potential

	Peak thrombin generation potential		
	B	CI	p
Active smoking	23.9	− 9.6–57.3	0.2
logPLT	272.1	125.5–418.6	< 0.001
logWBC	3.1	− 130.9–137.1	0.9
logCrea	− 81.2	− 236.9–74.4	0.3
logHsCRP	54.8	25–84.7	< 0.001
logHbA1c	346.2	106.2–586.2	0.005

Using the AUC as parameter of thrombin generation potential, increasing BMI, platelet count and HbA1c were associated with higher levels of thrombin generation potential, whereas increasing serum creatinine was linked to lower AUC values in univariate analyses (all p < 0.05). In multivariate linear regression analysis, BMI, serum creatinine and HbA1c remained independently associated with the AUC of thrombin generation potential (Table 3).

**Table 3:** Regression coefficients (B), confidence intervals (CI), and p-values of multivariate linear regression analyses of body mass index (BMI), log-transformed platelet count (logPLT), log-transformed serum creatinine (logCrea) and log-transformed hemoglobin A1c (logHbA1c) for area under the curve (AUC) of thrombin generation potential

	AUC of thrombin generation potential		
	B	CI	p
BMI	23	1.1 to 44.9	0.04
logPLT	677.7	− 83.8 to 1439.2	0.08
logCrea	− 1149.2	− 1970 to − 328.6	0.006
logHbA1c	1783.8	498.1 to 3069.6	0.007

**Fig. 1:**
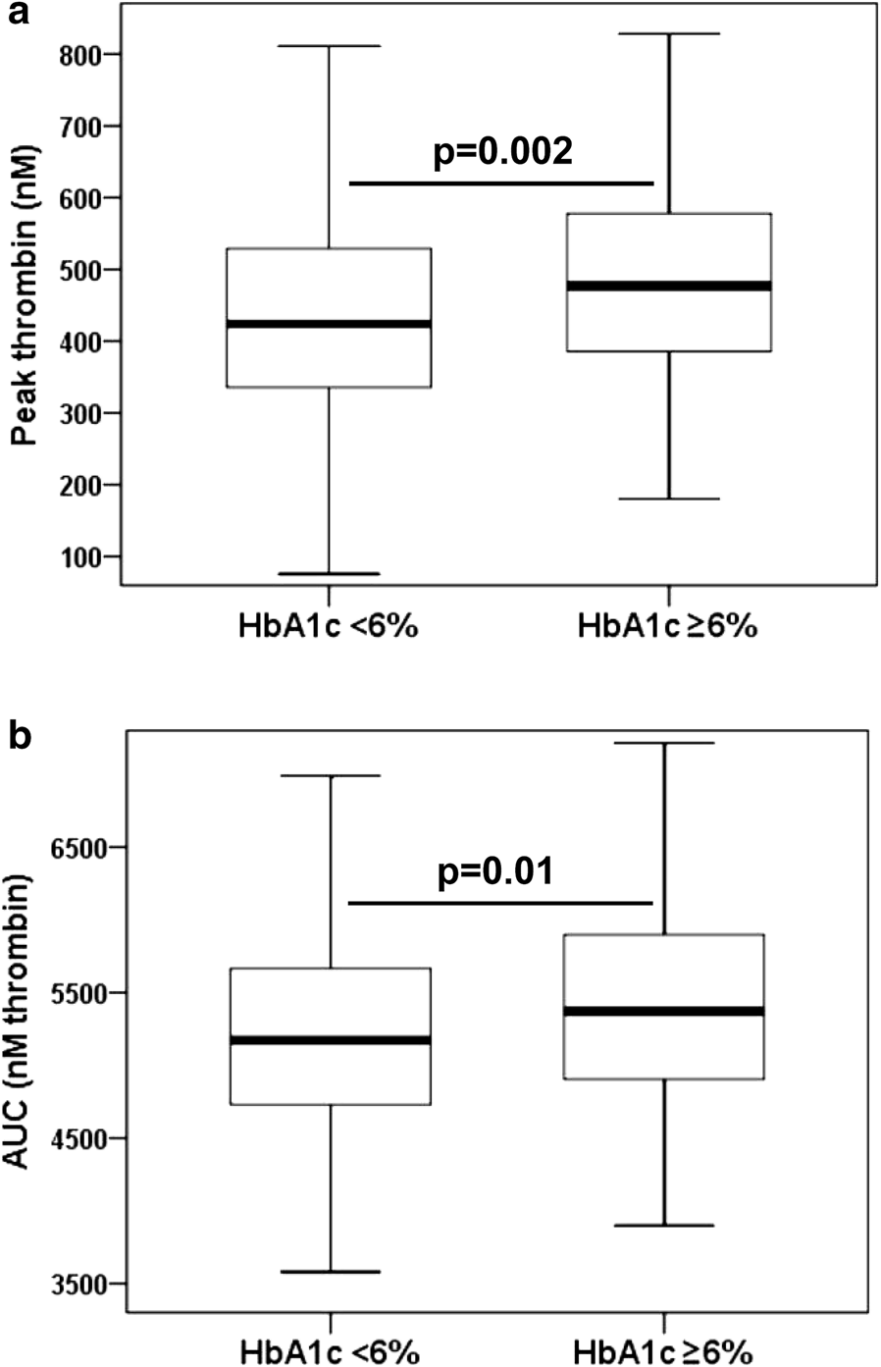
**a** Peak thrombin generation potential (peak thrombin) and **b** area under the curve of thrombin generation potential (AUC) in patients with normal (< 6%) and elevated (≥ 6%) hemoglobin A1c (HbA1c). The boundaries of the box show the lower and upper quartile of data, the line inside the box represents the median. Whiskers are drawn from the edge of the box to the highest and lowest values that are outside the box but within 1.5 times the box length

In a second step, we stratified the study population in patients with normal (< 6%) and elevated (≥ 6%) HbA1c levels according to a common definition of prediabetes [26, 27]. Those with elevated HbA1c were considered as patients with impaired glucose metabolism [28]. HbA1c was available for 301 patients (95.6%) of the study population, and with use of the above-mentioned cut-off value, normal and elevated HbA1c levels were seen in 156 (51.8%) and 145 (48.2%) patients, respectively. As expected, patients with HbA1c ≥ 6% had a higher BMI than patients with HbA1c < 6% (Table 1). Patients with elevated HbA1c had significantly higher peak thrombin generation potential and AUC of thrombin generation potential than patients with normal HbA1c (peak thrombin generation potential: 476.9 nM [385.8–577.9 nM] vs. 423.9 nM [335.8–529.5 nM], p = 0.002; AUC of thrombin generation potential: 5371.8 nM thrombin [4903–5899 nM thrombin] vs. 5172.5 nM thrombin [4731.8–5664.7 nM thrombin], p = 0.01; Fig. 1a, b). In multivariate linear regression analyses, elevated HbA1c remained independently associated with increased peak thrombin generation potential and AUC of thrombin generation potential (Tables 4, 5).

**Table 4:** Regression coefficients (B), confidence intervals (CI), and p-values of multivariate linear regression analyses of active smoking, log-transformed platelet count (logPLT), log-transformed white blood cell count (logWBC), log-transformed serum creatinine (logCrea), log-transformed high-sensitivity C-reactive protein (logHsCRP) and elevated hemoglobin A1c (HbA1c ≥ 6%) for peak thrombin generation potential

	Peak thrombin generation potential		
	B	CI	p
Active smoking	24	− 8.88 to 56.88	0.2
logPLT	271.3	126.53 to 416.15	< 0.001
logWBC	9.9	− 121.32 to 141.12	0.9
logCrea	− 117.7	− 272.44 to 37.12	0.1
logHsCRP	55.7	26.24 to 85.24	< 0.001
Elevated HbA1c	61.5	30.05 to 92.97	< 0.001

**Table 5:** Regression coefficients (B), confidence intervals (CI), and p-values of multivariate linear regression analyses of body mass index (BMI), log-transformed platelet count (logPLT), log-transformed serum creatinine (logCrea) and elevated hemoglobin A1c (HbA1c ≥ 6%) for area under the curve (AUC) of thrombin generation potential

	AUC of thrombin generation potential		
	B	CI	p
BMI	25.2	3.43 to 46.89	0.02
logPLT	698.6	− 64.18 to 1461.35	0.07
logCrea	− 1291.6	− 2118.38 to − 464.83	0.002
Elevated HbA1c	213.9	40.68 to 387.11	0.02

Prothrombin time, activated partial thromboplastin time, fibrinogen and D-dimer levels were similar in patients without and with elevated HbA1c (Table 6; all p ≥ 0.1).

**Table 6:** Coagulation parameters in patients with normal (< 6%) and elevated (≥ 6%) hemoglobin A1c (HbA1c)

	HbA1c < 6%	HbA1c ≥ 6%	*p* value
Prothrombin time, %	106 (93–118)	109 (94–123)	0.3
aPTT, s	36.3 (33.9–40.6)	35.5 (33.5–37.3)	0.1
Fibrinogen, mg/dl	409 (354–478)	411 (363–498)	0.5
D-dimer, mg/l	0.7 (0.4–1.3)	0.7 (0.5–1.2)	0.8

Continuous data are given as median (interquartile range)

*aPTT* activated partial thromboplastin time

## Discussion

To the best of our knowledge, our study is the first to investigate predictors of thrombin generation potential in patients with atherosclerotic cardiovascular disease undergoing angioplasty and stenting. HbA1c was the only variable that was independently associated with both, peak thrombin generation potential and AUC of thrombin generation potential. In contrast, platelet count and hsCRP were only associated with peak thrombin generation potential, and BMI and serum creatinine were only associated with AUC of thrombin generation potential after adjustment for covariates by multivariate linear regression analyses. Patients with HbA1c ≥ 6% had significantly higher peak thrombin generation potential and AUC of thrombin generation potential than patients with HbA1c < 6%.

### Association of thrombin generation potential with impaired glucose metabolism

Beijers et al. assessed thrombin generation potential with a different assay in 744 individuals from a cohort study on glucose metabolism in the general population [29]. In line with our results, they reported higher peak thrombin generation potential and increased endogenous thrombin generation potential in those with impaired glucose metabolism. Tripodi et al. analyzed blood samples from 60 patients with type 2 diabetes and found a significantly increased peak thrombin generation potential compared to 60 age- and gender-matched healthy controls [30]. Based on these previous observations and our findings, it may be speculated that increased thrombin generation potential at least in part contributes to the higher risk of ischemic events in diabetics undergoing angioplasty and stenting [31, 32]. However, clinical data linking thrombin generation potential with adverse cardiovascular outcomes in diabetics are missing, so far. Moreover, it remains to be established if antidiabetic therapy besides lowering blood glucose levels also affects thrombin generation potential. In our study, 11 patients with type 2 diabetes had HbA1c levels < 6% due to antidiabetic therapy (Table 1). These patients had numerically lower peak thrombin generation potential and AUC of thrombin generation potential than patients with HbA1c ≥ 6% (peak thrombin generation potential: 450.2 nM [397–522.5 nM] vs. 476.9 nM [385.8–577.9 nM]; AUC of thrombin generation potential: 5266.3 nM thrombin [4309–5658.8 nM thrombin] vs. 5371.8 nM thrombin [4903–5899 nM thrombin]; both p > 0.05).

Previous studies showed that subclinical and advanced atherosclerosis is associated with increased thrombin generation potential [33–35]. In line with these data, we found significantly higher peak thrombin generation potential and AUC of thrombin generation potential in patients undergoing angioplasty and stenting compared to 100 healthy individuals. Due to overall increased thrombin generation potential, the presence of macrovascular disease may abrogate the differences of thrombin generation potential between patients without and with impaired glucose metabolism. However, our findings of increased thrombin generation potential in patients with HbA1c ≥ 6% within a study population undergoing angioplasty and stenting, suggest that impaired glucose metabolism remains associated with thrombin generation potential even in advanced atherosclerotic cardiovascular disease.

### Potential underlying mechanisms

The detailed mechanisms leading to increased thrombin generation potential in patients with impaired glucose metabolism remain unclear. Since pronounced thrombin generation has been reported in obesity [36], the higher BMI in patients with elevated HbA1c in our study may partially explain their increased thrombin generation potential compared to those with normal HbA1c levels [29]. Moreover, it has previously been suggested that low-grade inflammation may enhance thrombin generation potential [29]. Indeed, hsCRP was independently associated with peak thrombin generation potential in our cohort. However, after adjusting for BMI and hsCRP in multivariate linear regression analyses, HbA1c ≥ 6% was still significantly associated with both parameters of thrombin generation potential thereby indicating that additional factors might play a role. Indeed, Rusak et al. recently showed that the incubation of platelets with glucose results in an increased mean platelet volume, more pronounced collagen-induced platelet aggregation, secretion, platelet-dependent thrombin generation and phosphatidylserine expression, thereby suggesting that hyperglycemia itself exerts procoagulatory effects [37]. In line with these findings, Soma et al. reported a higher expression of platelet surface receptors and enhanced platelet activation in type 2 diabetics compared to healthy individuals [38]. Besides hyperglycemia and increased endogenous thrombin generation, insulin receptor substrate-1 polymorphisms may be responsible for high on-treatment platelet reactivity in diabetes. In detail, carriage of the G allele of rs13431554 in the insulin receptor substrate-1 gene was associated with a hyperreactive platelet phenotype in diabetics with coronary artery disease [39]. Pretorius et al. revealed that the clotting of plasma from patients with type 2 diabetes is amyloid in nature and potentially induced by bacterial lipopolysaccharides [40]. Their findings suggest a microbial component in the development of type 2 diabetes. If so, lipopolysaccharide-binding protein might become a treatment option for diabetes and a way to prevent the associated coagulopathy.

### Association of thrombin generation potential with chronic kidney disease, inflammation and obesity

Interestingly, we observed decreasing peak thrombin generation potential and AUC of thrombin generation potential with increasing serum creatinine in univariate analyses. Furthermore, the association between AUC of thrombin generation potential and serum creatinine remained significant after adjustment for covariates in multivariate regression analysis, suggesting that thrombin generation potential decreases with worsening renal function. Thus, the increased bleeding risk in chronic kidney disease may be partially due to impaired thrombin generation [41, 42].

Our observation that platelet count and hsCRP were only associated with peak thrombin generation potential, and BMI and serum creatinine were only associated with AUC of thrombin generation potential after multivariate linear regression analyses may be explained by the underlying conditions: While platelet count and hsCRP can be considered as acute phase parameters reflecting inflammation, obesity and renal insufficiency are chronic comorbidities.

### Limitations

A limitation of our study is the lack of clinical outcome data. Moreover, we did not include matched patients without percutaneous intervention and we did not assess thrombin generation potential prior to angioplasty and stenting. Therefore, we cannot exclude an influence of the angioplasty procedure on thrombin generation potential. However, since angioplasty was performed in all patients and thrombin generation potential was assessed 1 day after the percutaneous intervention in all patients, it is unlikely that the angioplasty procedure affected the association between impaired glucose metabolism and thrombin generation potential.

## Conclusion

Impaired glucose metabolism is associated with increased thrombin generation potential in patients undergoing angioplasty and stenting for cardiovascular disease. Further studies are warranted to reveal potential clinical implications of these findings and to investigate if antidiabetic therapy decreases thrombin generation potential.

## Abbreviations

AUC: area under the curve
HbA1c: hemoglobin A1c
hsCRP: high-sensitivity C-reactive protein
VTE: venous thromboembolism
WBC: white blood cell count
